# Sensory processing sensitivity in adolescents reporting chronic pain: an exploratory study

**DOI:** 10.1097/PR9.0000000000001053

**Published:** 2023-01-06

**Authors:** Helen Koechlin, Carolina Donado, Cosima Locher, Joe Kossowsky, Francesca Lionetti, Michael Pluess

**Affiliations:** a Division of Psychosomatics and Psychiatry, University Children's Hospital Zurich, Zurich, Switzerland; b Department of Anesthesiology, Critical Care and Pain Medicine, Harvard Medical School, Boston Children's Hospital, Boston, MA, USA; c Department of Consultation-Liaison Psychiatry and Psychosomatic Medicine, University Hospital Zurich, University of Zurich, Zurich, Switzerland; d Faculty of Health, University of Plymouth, Plymouth, United Kingdom; e Department of Neuroscience, Imaging and Clinical Sciences, G. d'Annunzio University of Chieti-Pescara, Chieti - Pescara, Italy; f Department of Biological and Experimental Psychology, Queen Mary University, London, United Kingdom

**Keywords:** Sensory processing sensitivity, Chronic pain, Adolescents, Emotion regulation, Quality of life

## Abstract

**Introduction:**

Sensory processing sensitivity (SPS) describes a genetically influenced trait characterized by greater *depth* of information *processing*, lower sensory threshold, and ease of overstimulation. It is hypothesized that SPS plays a crucial role in the context of chronic pain.

**Objectives:** This exploratory study examined SPS as a correlate of pain intensity and pain-related disability in a sample of adolescents reporting chronic pain.

**Methods:**

Adolescents reporting chronic pain were contacted through social media and through specialized pain clinics. Participants completed online questionnaires on their levels of SPS, pain features, emotion regulation, and quality of life. A series of analysis of variances (ANOVAs) were calculated to detect differences between 3 SPS groups (ie, high, medium, and low sensitivity) regarding emotion regulation, quality of life, and pain features. Multiple linear regressions were conducted to predict pain intensity, pain-related disability, and quality of life.

**Results:**

In total, 103 participants completed the survey (68.9% female, M_age_ 17.9). Back pain was the most frequently reported pain location. Proportion of highly sensitive individuals was large (45.68%). The ANOVA revealed significant differences between sensitivity groups related to quality-of-life subscales, namely, for physical (F(2, 100) = 7.42, *P* < 0.001), emotional (F(2, 100) = 6.11, *P* < 0.001), and school functioning (F(2, 100) = 3.75, *P* = 0.03). High sensitivity was not predictive of pain but of health-related quality of life.

**Conclusions:**

Our results indicate that SPS is an important and prevalent characteristic to consider in the context of chronic pain in adolescents, specifically regarding the quality of life.

## Abstract 2

Supplemental Digital Content is Available in the Text.

Sensory processing sensitivity, a genetically influenced trait characterized by lower sensory threshold, might be a prevalent characteristic of adolescents reporting chronic pain.

## 1. Introduction

Up to 1 in 4 children will have an episode of chronic pain, ie, pain lasting for 3 months or longer, according to estimates based on the available literature.^27^ Chronic pain not only is linked to significant psychological, physical, and social concerns for affected children and their families^14,36^ but also places an enormous burden on health care systems and ranks among the most expensive pediatric health problems in the United States^20^ and in Europe.^47,50^ The International Association for the Study of Pain (IASP) defines pain as an “unpleasant sensory and emotional experience associated with, or resembling that associated with, actual or potential tissue damage,”^45^ which points to the biopsychosocial nature of chronic pain.^17^ Chronic pain is associated with a range of negative emotions (eg, anger, anxiety, depressed mood),^39^ difficulties with emotion regulation,^28^ and also often comorbid with anxiety and depressive disorders.^49,52^ In addition, in a study of pediatric patients with chronic pain seeking outpatient pain management services, participants reported lower health-related quality of life than the general population,^18^ with scores significantly lower than those reported by pediatric patients with rheumatologic conditions^57^ and cancer-related pain.^56^

Currently, it is difficult to reliably predict who is at risk for the development of pediatric chronic pain, although a range of psychological, behavioral, and neurobiological factors are discussed in the literature.^24,33,34,43^ Pain vulnerability is shaped by a complex interplay of sensory, environmental, psychological, and pain regulatory risk factors.^58^ Within this interplay, 1 important component is central sensitization: As a result of suspected genetic susceptibility, repeated trauma, infections, and inflammation, the central nervous system might become overly effective in (or sensitive to) transmitting nociceptive stimuli and less effective in inhibiting them.^12,35,62^ Central sensitization leads to lowered sensory threshold: Due to increases in synaptic efficacy of nociceptive stimulus transmission and reduction in inhibition processes, the pain response to noxious stimuli is enhanced. As a consequence, low-threshold sensory inputs can more easily activate the pain circuit.^62^ Pain that arises from central sensitization is defined as nociplastic pain, and hypersensitivity to environmental stimuli such as light or sound is suggestive of nociplastic pain.^16^

Interestingly, high sensitivity to external stimuli (such as bright lights and loud noises) is also one of the key characteristics of *Sensory Processing Sensitivity* (SPS).^29^ Sensory processing sensitivity describes a genetically influenced temperament trait characterized by greater depth of information processing, increased emotional reactivity, and tendency for overstimulation.^3^ Individuals high in SPS are more prone to “pause to check” in a novel situation, are more sensitive to subtle stimuli, employ deeper or more complex cognitive processing strategies, show a lower threshold to sensory inputs, and a stronger positive and negative emotional reactivity.^1,2^ Empirical data suggest that a significant minority of the population, ranging between 25% to 30%, are highly sensitive to environmental influences.^7,40^

The framework of *Differential Susceptibility*^7^ states that more sensitive individuals differ from less sensitive peers not only in their response to environmental adversity (as implied by the traditional diathesis-stress framework) but also in response to positive aspects of the environment (eg, social support, sensitive parenting).^41^ In order to describe more and less sensitive individuals,^9^ the *orchid-dandelion* metaphor has been adopted. In this metaphor, *orchids* represent the more sensitive individuals who do exceptionally well under ideal conditions but also struggle more under poor circumstances, and *dandelions* represent those who are generally less sensitive, considered resilient and can grow anywhere. However, a more recent study has challenged this understanding, as Lionetti et al.^29^ found 3 rather than 2 groups in their sample of more than 900 adults and, in accordance with the botanic metaphor, have referred to the group characterized by medium sensitivity as *tulips*. The distribution of the 3 groups was approximately 30% for the high and low SPS, whereas 40% were characterized by medium sensitivity, suggesting that SPS is a normally distributed, continuous trait along which people fall into 3 groups. A very similar distribution of sensitivity has been found in more than 3000 children and adolescents aged 8 to 19 years: Approximately 35% of children and adolescents belonged to a low sensitive group, 41% to 46% to a medium sensitive group, and 21% to 23% to a highly sensitive group.^42^

Sensory processing sensitivity in adults can be measured with the *Highly Sensitive Person* scale (HSP^1^) that has been adapted and validated for children and adolescents (*Highly Sensitive Child Scale* [HSC]^42^). In psychometric evaluations, 3 factors of the HSC emerged: *aesthetic sensitivity* (AES; eg, being deeply moved by music and arts), *ease of excitation* (EOE; eg, negative response to having a lot going on), and *low sensory threshold* (LST; eg, reaction to bright lights and loud noises).^42,51^ Furthermore, the HSC also reflects a general sensitivity factor, calculated as the total score of the scale.^42^ Importantly, sensitivity correlates with several affective and personality traits: A recent meta-analysis on the relationship between SPS and affect found a moderate effect size for negative affect in children and adults, whereas an association with positive affect could only be found in children and was driven mainly by the AES subscale.^30^ As the associations were small to moderate across the meta-analysis, it can be concluded that SPS is a distinct construct that is related to (especially) negative affect but does not fully overlap with it.^3,30^

Given the potential theoretical links between pain vulnerability and SPS, we set out to explore the role of SPS in a sample of adolescents reporting chronic pain. In addition to SPS, we also collected data on pain features, quality of life, and emotion regulation. The choice of constructs other than SPS is in line with the results of a recent Delphi poll that captured providers', patients', and parents' perspectives about core outcome domains for pediatric chronic pain intervention studies^37^ and has been adapted for our purpose. The developmental stage of late adolescence has often been overlooked in research focusing on factors that might impact pain and pain-related outcomes.^38^ Therefore, we focused on this specific age group in our study. As this was an exploratory study, we were interested in examining (1) whether HSC subscales could help predict pain intensity and pain-related disability in a sample of adolescents reporting chronic pain, and (2) how quality of life and use of emotion regulation strategies differed between groups of high, medium, and low sensitivity. To the best of our knowledge, this is the first undertaking to study SPS in the context of pediatric pain.

## 2. Materials and methods

We conducted an online survey among older adolescents reporting chronic pain containing several questionnaires.

### 2.1. Sample and procedure

Participants had to be between 17 and 19 years of age and experienced chronic or recurrent pain for at least 3 months. The survey was created using an online tool at the University of Basel, Switzerland. The Ethics Committee of the Faculty of Psychology, University of Basel, approved the study (ID: 028-19-1). Participants were recruited through forums for patients with chronic pain, with flyers sent to schools and specialized pain clinics in Switzerland, and posted on social media. A QR-Code on the flyers brought participants directly to the online survey. The survey was available in German and English. The completion of the survey took approximately 25 minutes, and participants were reimbursed with a 10 Swiss Francs voucher from a large Swiss online shop.

### 2.2. Questionnaires

Participants completed a set of questions regarding their age, gender (ie, drop-down menu with “female,” “male,” “non-binary,” and “other”), living situation, education, pain diagnosis (if they have received one), pain duration, pain location, other diagnoses and symptoms, and current treatment of pain. In addition, participants rated their average pain intensity, pain-related disability, and pain-related distress over the past weeks (as recommended in the context of chronic pain^5,54^), using a visual analogue scale (VAS), presented as a horizontal line and ranging from 0 to 10, with 0 indicating no pain/disability/distress and 10 indicating worst pain/disability/distress. Participants were asked to visualize their pain on the horizontal line to described their average pain/disability/distress.^54^

#### 2.2.1. Sensitivity

Participants' sensitivity was measured using the HSC,^42^ a 12-item self-report questionnaire that has been validated in children and adolescents aged 8 to 19 years. Participants are asked to rate each item on a scale from 1 (=not at all) to 7 (=extremely). The HSC is composed of a total score and 3 factors: ease of excitation (EOE; eg, “I get nervous when I have to do a lot in little time”), aesthetic sensitivity (AES; eg, “I love nice smells”), and low sensory threshold (LST; “I don't like watching TV programs that have a lot of violence in them”). Higher scores indicate higher sensitivity. Cronbach α was 0.819 for the EOE subscale (5 items), 0.589 for the AES subscale (4 items), 0.581 for the LST subscale (3 items), and 0.826 for the HSC total score in the current sample. This is slightly different from the original study,^42^ which found acceptable internal consistencies for all subscales (but still slightly lower α for AES). This might be due to the fact that all participants in our study reported chronic pain, whereas in previous studies, healthy adolescents were recruited from the general population.

#### 2.2.2. Pain-related disability

The Functional Disability Inventory for children (FDI)^60^ consists of 15 items that assess difficulties in performing daily activities. The FDI asks about physical functioning and disability (eg, “in the last few days, have you had any physical trouble or difficulties … walking to the bathroom? Eating regular meals?”) in youth with chronic pain. Each item is rated on a 5-point Likert scale ranging from *no problem* to *not possible*. The FDI is well-established and commonly used in pediatric chronic pain samples. Higher scores indicate higher disability. Cronbach α was 0.905 in this sample.

#### 2.2.3. Emotion regulation

The Emotion Regulation Questionnaire (ERQ),^21^ a 10 items self-report questionnaire, is designed to assess individual differences in the habitual use of 2 emotion regulation strategies, namely, *cognitive reappraisal* (eg, “When I'm faced with a stressful situation, I make myself think about it in a way that helps me stay calm”) and *expressive suppression* (eg, “I keep my emotions to myself”). Items are rated on a scale from 1 (=strongly disagree) to 7 (=strongly agree). Higher scores indicate more habitual use of the respective emotion regulation strategy. Cronbach α was 0.718 for the cognitive reappraisal subscale (6 items) and 0.523 for the expressive suppression subscale (4 items) in this sample. Low Cronbach α for the expressive suppression subscale was mainly driven by the item “When I'm feeling *positive* emotions, I am careful not to express them.” This item might have been misunderstood in the context of the other questions for this subscale, which are all related to emotions in general or to negative emotions specifically.

#### 2.2.4. Quality of life

The Pediatric Quality of Life Inventory (PedsQL)^55^ is a 23-item, multidimensional, self-report questionnaire for children and adolescents. Items are rated on a scale from 0 (=never a problem) to 4 (=almost always a problem). The questionnaire asks about physical functioning (eg, “It is hard for me to run”), emotional functioning (eg, “I feel sad or blue”), social functioning (eg, “It is hard to keep up with my peers”), and school functioning (eg, “It is hard to pay attention in class”) in the past 1 month. Higher scores indicate better quality of life. Cronbach α was 0.825 for physical functioning (8 items), 0.847 for emotional functioning (5 items), 0.781 for social functioning (5 items), and 0.817 for school functioning (5 items) in this sample. A physical health score (ie, the physical functioning subscale) and a psychosocial health summary score (ie, mean of all items of the emotional, social, and school functioning subscales, Cronbach α = 0.865) can be created and were used in the multiple linear regression models.

### 2.3. Statistical analyses

We used descriptive statistics to summarize participant characteristics. We used density plots to explore HSC values distribution, both of the total score and the 3 subscales EOE, AES, and LST.

We categorized participants based on their sensitivity into 3 groups (ie, low, medium, and high sensitivity), applying previously reported preliminary cutoff scores.^42^ Differences between the 3 groups in pain-related disability, the 4 domains of quality of life (ie, physical functioning, emotional functioning, social functioning, and school functioning), and the 2 emotion regulation strategies (ie, expressive suppression and cognitive reappraisal) were calculated using a series of 1-way analysis of variance (ANOVA). Additional ANOVAs comparing the top and bottom 30% of the sample with the 40% in the middle can be found in the Supplemental digital content (available at http://links.lww.com/PR9/A181).

We then conducted a stepwise approach in separate multiple linear regression models for the outcomes pain-related disability (ie, FDI total score) and pain intensity (ie, VAS average pain intensity). Participants' age, gender, and quality of life (PedsQL) summary scores (physical health score and psychosocial health summary score) were included as cofounders. The HSC total scores and the 2 emotion regulation strategies (ie, cognitive reappraisal and expressive suppression) were included as coefficients. We then replaced the HSC total score with its subscales EOE, AES, and LST to check for more specific associations.

For our analyses with 3 groups and 5% error level, we estimated that a sample size of N = 114 would provide 75% power to detect a medium effect (based on^13^—which, however, used an adult sample).

Analyses were conducted in R (version 4.0.4),^44^ using the RStudio development (version 1.1463)^46^ and SPSS (version 28.0.1.1).^25^

## 3. Results

In total, 256 adolescents clicked on the link to the survey. Of those, 103 did not consent to participate and stopped the survey before answering any questions. An additional 49 adolescents consented to participate but did not complete the survey—half of them stopped answering questions on the first page of the survey, when asked about their sociodemographic background. In total, 103 participants completed the whole survey and were included in the analyses. Of those, 68.9% identified as female, 31.1% as male. Mean age was 17.9 years (SD = 1.43), 75.7% were still going to school and 30.1% were in training. Most participants lived with their parents (91.3%), and 48.5% were currently receiving some type of therapy for their pain, with physiotherapy being the most common (mentioned by 36.9% of the total sample; multiple answers possible). The pain location mentioned most often was back pain (42%), followed by headache (33%), neck pain (27%), pain in the legs (22%), arms (8%), and pain in the stomach (7%; multiple answers possible). Mean pain duration was 22 months (SD = 19.8). Mean pain intensity was rated as a 5.8 (SD = 1.9), indicating moderate pain.^10^ This is comparable to other youth samples with chronic pain.^6,8^ Mean pain-related interference was 6.0 (SD = 2.3), and mean pain-related distress was rated as 5.3 (SD = 3.0). See Table 1 for demographics of the sample. Correlations between variables can be found in Table 2.

**Table 1:** Sample characteristics.

	Overall
n	103
Gender = male, n (%)	32 (31.1)
Age (mean [SD])	17.92 (1.43)
Going to school (%)	78 (75.7)
Apprenticeship (%)	31 (30.1)
Housing situation	
Living with parents (%)	94 (91.3)
Living with siblings (%)	59 (57.3)
Pain location*	
Head (%)	33 (32.0)
Stomach (%)	7 (6.8)
Arms (%)	8 (7.8)
Legs (%)	22 (21.4)
Back (%)	42 (40.8)
Neck (%)	27 (26.2)
Pain intensity (mean [SD])	5.87 (1.92)
Pain-related interference (mean [SD])	6.03 (2.31)
Pain-related distress (mean [SD])	5.26 (2.97)
Current treatment*	
Physiotherapy (%)	38 (36.9)
Medication (%)	19 (18.4)
Occupational therapy (%)	3 (2.9)
Alternative medicine (%)	14 (13.6)
Psychotherapy (%)	14 (13.6)
FDI total score (mean [SD])	15.17 (10.30)
Quality of life (PedsQL)	
Physical functioning (mean [SD])	62.77 (18.88)
Emotional functioning (mean [SD])	52.52 (23.24)
Social functioning (mean [SD])	75.34 (19.53)
School functioning (mean [SD])	58.79 (22.66)
Emotion regulation (ERQ)	
Cognitive reappraisal (mean [SD])	4.87 (1.26)
Expressive suppression (mean [SD])	4.39 (1.13)
Sensitivity (HSC)	
Total score (mean [SD])	4.60 (1.02)
Ease of excitation (mean [SD])	4.45 (1.34)
Aesthetic sensitivity (mean [SD])	5.53 (1.01)
Low sensory threshold (mean [SD])	3.80 (1.44)

* Multiple responses possible.

ERQ, Emotion Regulation Questionnaire; FDI, Functional Disability Index; HSC, highly sensitive child; PedsQL, Pediatric Quality of Life Inventory.

**Table 2:** Correlation matrix.

	1	2	3	4	5	6	7	8	9	10	11	12	13
1. Age	—												
2. Pain intensity	<0.1	—											
3. Pain interference	<0.1	0.585**	—										
4. Pain-related distress	<0.1	0.523**	−0.514**	—									
5. FDI total score	<0.1	0.418**	0.479**	0.550**	—								
6. HSC total score	0.139	<0.1	0.147	0.231*	0.277**	—							
7. HSC EOE score	0.109	<0.1	0.127	0.230	0.237*	0.929**	—						
8. HSC AES score	<0.1	<0.1	<0.1	<0.1	<0.1	0.651**	0.531**	—					
9. HSC LST score	0.195*	<0.1	0.260**	0.311**	0.321**	0.821**	0.652**	0.374**	—				
10. ERQ reappraisal score	<0.1	<0.1	−0.123	−0.211*	−0.334**	−0.195*	−0.228*	<0.1	−0.137	—			
11. ERQ suppression score	0.183	<0.1	−0.191	<0.1	−0.193*	−0.164	−0.159	−0.225*	<0.1	0.381**	—		
12. PedsQL psychosocial health summary score	<0.1	−0.456**	−0.397**	−0.533**	−0.592**	−0.359**	−0.379**	<0.1	−0.328**	0.339**	0.135	—	
13. PedsQL physical functioning	<0.1	−0.398**	−0.437**	−0.395**	−0.753**	−0.390**	−0.355**	−0.257**	−0.349**	0.244*	0.143	0.561**	—

*P* values <0.05 are marked in bold.

**P < 0.01 (2 tailed). *P < 0.05 (2 tailed).

AES, aesthetic sensitivity; EOE, ease of excitation; ERQ, Emotion Regulation Questionnaire; FDI, Functional Disability Inventory; HSC, highly sensitive child scale; LST, low sensory threshold; PedsQL, Pediatric Quality of Life Inventory.

### 3.1. Differences between sensory processing sensitivity groups

Participants were divided into groups based on their HSC ratings. Cutoff scores for the 3 groups were based on a previous article examining the validity of the HSC scale in a sample of healthy adolescents in the United Kingdom.^42^ When applying these cutoff scores, the high sensitivity group (ie, values above 4.65) consisted of N = 47 (45.63%), the medium group (ie, values between 3.65 and 4.65) consisted of N = 41 (39.81%), and the low sensitivity group (ie, values equal to or lower than 3.64) consisted of N = 15 (14.56%). No differences in pain-related disability or emotion regulation strategies (ie, expressive suppression and cognitive reappraisal) were found between the sensitivity groups. For quality of life, significant differences between the groups emerged for physical functioning, with a mean of 58.99 (SD = 18.68) for the high sensitivity group, M = 63.34 (SD = 17.73) for the medium sensitivity group, and M = 73.12 (SD = 19.7) for the low sensitivity group. For the emotional functioning subscale, mean was 45.74 (SD = 22.8) for the high sensitivity group, M = 55.85 (SD = 21.6) for the medium sensitivity group, and M = 64.67 (SD = 23.5) for the low sensitivity group. For school functioning, the high sensitivity group reported M = 53.4 (SD = 23.88), the medium sensitivity group M = 60.98 (SD = 22.11), and the low sensitivity group M = 69.67 (SD = 15.3). No significant differences were found for social functioning. Table 3 provides values for all variables by sensitivity group, and Table 4 provides ANOVA results; see the Supplemental digital content (available at http://links.lww.com/PR9/A181) for results of sensitivity groups based on the top and bottom 30% of the sample with the 40% in the middle.

**Table 3:** Characteristics of sensitivity groups.

Characteristics of SPS groups, M(SD)				
	High sensitivity	Medium sensitivity	Low sensitivity	Significant at *P* < 0.05
Pain intensity	6.06 (2.00)	5.73 (1.88)	5.67 (1.88)	—
Pain-related interference	6.30 (2.50)	6.02 (2.12)	5.20 (2.21)	—
Pain-related distress	6.06 (3.02)	4.44 (2.83)	5.00 (2.73)	0.034
FDI total score	16.20 (10.93)	15.22 (10.36)	11.87 (7.61)	—
PedsQL physical functioning	58.99 (18.68)	63.34 (17.73)	73.12 (19.70)	0.038
PedsQL emotional functioning	45.74 (22.80)	55.85 (21.60)	64.67 (23.50)	0.010
PedsQL social functioning	72.23 (21.80)	77.68 (16.60)	78.67 (19.32)	—
PedsQL school functioning	53.40 (23.88)	60.98 (22.11)	69.67 (15.30)	0.037
Cognitive reappraisal	4.65 (1.23)	5.08 (1.18)	5.02 (1.55)	—
Expressive suppression	4.23 (1.05)	4.50 (1.07)	4.63 (1.50)	—
HSC EOE score	5.52 (0.92)	3.96 (0.60)	2.43 (0.60)	<0.001
HSC AES score	6.03 (0.69)	5.38 (0.73)	4.35 (1.40)	<0.001
HSC LST score	4.77 (1.22)	3.33 (0.87)	2.02 (0.90)	<0.001

For cognitive reappraisal, expressive suppression, and the HSC total and subscale scores: higher scores indicate higher use of emotion regulation strategy or higher sensitivity, respectively.

AES, aesthetic sensitivity; EOE, ease of excitation; FDI, Functional Disability Inventory (higher scores indicate greater functional disability); HSC, highly sensitive child scale; LST, low sensory threshold; PedsQL, Pediatric Quality of Life Inventory (higher scores indicate better quality of life).

**Table 4:** Analysis of variance results.

Sensitivity groups (df = 2)				
	Sum Sq	Mean Sq	F value	*P* (>F)
FDI total score	213	106.4	1.004	0.37
Emotion regulation				
Cognitive reappraisal	4.55	2.27	1.43	0.24
Expressive suppression	2.42	1.21	0.95	0.39
Quality of life				
Physical functioning	2298	1149.1	3.37	0.04*
Emotional functioning	4826	2413.1	4.80	0.01*
Social functioning	844	422.2	1.11	0.33
School functioning	3334	1666.8	3.40	0.04*

Significance: *0.05. Significant results are highlighted in bold.

FDI, Functional Disability Inventory.

Due to differences in the quality of life between the sensitivity groups, we included the 2 quality of life summary scores (psychosocial health summary score and physical health score) in subsequent analyses.

### 3.2. Prediction of pain-related disability

In the initial multiple linear regression, neither age nor gender was statistically significant. They were therefore removed from the subsequent analyses. The model using the HSC total score showed significant associations between the PedsQL psychosocial health summary score and physical health score, and the total FDI score (F(5,96) = 32.75, *P* < 0.0001). The *P* value of the F-statistic (F(7, 94) = 24.28) for the model, including HSC subscales, was highly significant (*P* < 0.001). The estimated effects (β) were significant for the PedsQL psychosocial health summary score (β = −0.13, *P* = 0.008) and physical health score (β = −0.33, *P* < 0.001). Highly sensitive child scale subscales LST and EOE were borderline significant. All estimated effects and the model summary can be found in Table 5.

**Table 5:** Multiple regressions.

Pain-related disability (FDI total score)				
	Estimated effect (β)	SE	*t*	*P*
Intercept	56.83	5.69	9.99	<0.001***
Ease of excitation	−1.27	0.68	−1.87	0.0643
Aesthetic sensitivity	−0.73	0.76	−0.96	0.3375
Low sensory threshold	1.09	0.56	1.94	0.0556
ERQ reappraisal	−0.79	0.55	−1.45	0.1508
ERQ suppression	−0.83	0.60	−1.38	0.1699
PedsQL psychosocial health summary score	−0.13	0.05	−2.68	0.0088**
PedsQL physical health score	−0.33	0.04	−8.04	<0.001***
Model summary: F(7, 94) = 25.28, *P* < 0.001; adjusted *R*^2^ = 0.627.				

Pain intensity				
	Estimated effect (β)	SE	*t*	*P*
Intercept	10.44	1.54	6.76	<0.001***
Ease of excitation	−0.08	0.18	−0.41	0.6809
Aesthetic sensitivity	−0.23	0.21	−1.11	0.2709
Low sensory threshold	−0.07	0.15	−0.47	0.6388
ERQ reappraisal	0.08	0.15	0.54	0.5922
ERQ suppression	0.06	0.16	0.40	0.6932
PedsQL psychosocial health summary score	−0.04	0.01	−3.18	0.0020**
PedsQL physical health score	−0.03	0.01	−2.53	0.0132*
Model summary: F(7, 94) = 5.175, *P* < 0.001, adjusted *R*^2^ = 0.224.				

SE, standard error; *t*, test statistic (2-sided *t* test); *P < 0.05, **P < 0.01, ***P < 0.001.

ERQ, Emotion Regulation Questionnaire; FDI, Functional Disability Inventory; PedsQL, Pediatric Quality of Life Inventory.

### 3.3. Prediction of pain intensity

In the initial multiple linear regression, neither age nor gender were statistically significant. Therefore, they were removed from the subsequent analyses. The HSC total score was marginally significantly associated with pain intensity, and PedsQL psychosocial health summary score and physical health score were statistically significant. When considering the HSC subscales, none of them were significant. The PedsQL psychosocial health summary score and the PedsQL physical health score both were significant (Table 5).

## 4. Discussion

To the best of our knowledge, this is the first study to explore SPS in a sample of adolescents reporting chronic pain. Previous research on SPS in healthy adolescents found 20% to 35% of the sample to be highly sensitive, 41% to 47% to be of medium sensitivity, and the remaining 25% to 35% to be of low sensitivity.^42^ Using the cutoff scores provided in their analysis, we found 45.63% of our sample to be highly sensitive, 39.81% to fall into the medium sensitivity group, and 14.56% to display low sensitivity. This points to a different distribution of sensitivity in this sample of adolescents reporting chronic pain compared with healthy adolescents from a previous study,^42^ which could be due to various reasons. In our sample, we found that LST scores were partially responsible for high sensitivity values. LST stands for *low sensory threshold*, which, in the context of pain, seems to be of particular relevance: In adults, studies have found individuals' sensory function, including pain perception and pain modulation, to be an important predictor for the development of chronic pain.^19,63^ Greater pain sensitivity could be a consequence of lower threshold for sensory input and in turn increase the risk for the development or maintenance of chronic pain. Accordingly, an experimental study comparing pain sensitivity in pediatric patients with chronic pain and healthy controls found lower pain threshold and tolerance in adolescents with chronic pain compared with healthy peers.^53^

In our sample, LST predicted neither pain intensity nor pain-related disability. This is surprising, given that a lower sensory threshold towards external stimuli might increase pain perception. One explanation for this is potentially altered interoception, ie, the ability to sense changes in physiological sensations from inside the body (including pain).^22,23^ Increased interoception—and hence heightened pain perception—has been suggested to be prevalent amongst individuals with chronic pain,^22,23^ and potentially even more so in highly sensitive individuals with low sensory thresholds. However, it is well known from the pain literature that pain is highly subjective^10^ and that chronic pain can be disabling for some people, whereas others live surprisingly well with it.^59^ Furthermore, pain intensity does not necessarily correspond to pain-related disability,^32^ although it clearly did in our sample.

Regarding emotion regulation, we found no significant differences due to sensitivity groups in the emotion regulation strategy cognitive reappraisal. This is in contrast to a recent study with adult participants that found difficulties in emotion regulation (indicated in their study by nonacceptance of emotional responses, lack of emotional awareness, and limited access to emotion regulation strategies) to be significantly correlated with the Highly Sensitive Person scale (the adult version of the HSC).^11^ In our sample, the lack of association might be due to the fact that we did not measure environmental variables. A recent study by Lionetti et al.^31^ found HSC to correlate with rumination (which is considered to be a regulatory strategy) only when the quality of the parenting was less than optimal. Another possible explanation is that in our sample, only minor variability of sensitivity scores was observed at the low end of the continuum because the majority of participants in our sample were highly sensitive.

More broadly, and in the context of pediatric pain, previous research also indicates that aspects of emotional functioning contribute to the pain experience; negative affect and its detrimental consequences have been studied extensively in the context of chronic pain.^15,26,61^ Likewise, fear of pain, negative expectations, pain catastrophizing, or overpredictions of pain play a significant role in the maintenance of pain.^4,48^ Living with chronic pain might therefore present with similarly high demands for emotion regulation^28^ across sensitivity groups.

For quality of life, our results showed differences between the sensitivity groups in all but one subscale of the measure, namely, for physical, emotional, and school functioning but not social functioning: Participants in the high sensitivity group consistently reported lower levels of quality of life, with the smallest (and nonsignificant) difference in the social functioning domain. By contrast, participants in the low sensitivity group revealed highest mean scores on all quality of life domains.

### 4.1. Limitations

Our study has several limitations. First, it is a cross-sectional and correlational design, the sample was self-selected, and we were not able to verify the information provided (eg, regarding diagnosis). Second, there was no control group without chronic pain who completed the same set of questionnaires; hence, we relied on a qualitative comparison with previously reported HSC data in healthy samples.^42^ Third, we did not assess environmental influences, such as familial context of participants. As highly sensitive individuals profit enormously from positive and supportive contexts, this might have moderated the influence of sensitivity on pain intensity and pain-related disability in some of the participants. Finally, our sample size was small, resulting in a low sensitivity group of just N = 15.

## 5. Conclusion and future directions

Future research should assess SPS as a potential risk factor for pain chronification and a factor that could be involved in the adjustment of adolescents reporting chronic pain. Importantly, in this context, factors that might moderate the association between SPS and pain, namely, the quality of the familial and social environment should be considered as well. Highly sensitive individuals tend to respond exceptionally well to interventions and hence assessing sensitivity in a clinical context could be relevant to better tailor intervention and prevention programs in a population that seems to score particularly high on sensitivity. Regarding quality of life, physical, emotional, and school functioning should be carefully assessed in the context of pediatric chronic pain—and high SPS. Highly sensitive adolescents might experience lower quality of life in those domains, putting them at risk for related problems, such as anxiety or depressive symptoms, or problems in school, suggesting that a better knowledge of the SPS trait and experience of pain can be relevant not only for practitioners but also for all caregivers who are part of children's everyday environment, such as teachers and parents.

## Disclosures

The authors have no conflict of interest to declare.

## Appendix A. Supplemental digital content

Supplemental digital content associated with this article can be found online at http://links.lww.com/PR9/A181.
